# Development and validation of SALT Triage method to facilitate the identification and classification of patients in Mass Casualty Incidents

**DOI:** 10.5249/jivr.v15i2.1681

**Published:** 2023-07

**Authors:** Ghasem Mehralian, Marzieh Pazokian, Yousof Akbari Shahrestanaki, Amir Salari, Amin Saberinia, Soheil Soltani

**Affiliations:** ^*a*^ Department of Medical Surgical Nursing, School of Nursing and Midwifery, Shahid Beheshti University of Medical Sciences, Tehran, Iran.; ^*b*^ Department of Pre-hospital Medical Emergencies, School of Paramedical Sciences, Qazvin University of Medical Sciences, Qazvin, Iran.; ^*c*^ Imam Khomeini Hospital Complex, Deputy of University Affairs, Tehran University of Medical Sciences, Tehran, Iran.; ^*d*^ Department of Emergency Medicine, School of Medicine, Kerman University of Medical Sciences, Kerman, Iran.; ^*e*^ Emergency Department of the Shohadaye Tajrish Hospital, Shahid Beheshti University of Medical Sciences, Tehran, Iran.

**Keywords:** Mass Casualty, Incident (MCI), Triage, Modification

## Abstract

**Background:**

Mass Casualty Incidents (MCIs) have caused great financial losses. These incidents are referred to a situation in which the number of casualties caused by the accident temporarily increases to such an extent that it is not possible to treat all these patients with the facilities and capacities available in the area. To offer fair and proportionate medical services to all patients, it is necessary to use a process called patient triage. This study aimed to modify the Sort Assess Lifesaving Intervention Treatment/transport (SALT) triage method to simplify the differentiation of patients from green from yellow and gray from red.

**Methods:**

This is a methodological study with a descriptive cross-sectional approach that by studying the SALT triage method and using the criteria defined in the Reference Standard, facilitates the identification of patients with a minor (Outpatient) and fatal injury (Expectant). Then, using two common and modified SALT triage methods, 100 simulated patients were triaged and the obtained data were evaluated and compared in terms of accuracy and speed.

**Results:**

The improvement made in the SALT triage method was able to reduce 22% of the total triage error of the first nurse and improved 18% in green, 43% in yellow, 15% in red, and 13% in the gray category. In the second nurse, this method was able to reduce 29% of the errors and in the category of green patients, 41%, yellow 47%, red was unchanged, and gray 38% improvement was observed. Furthermore, the average triage rate was 4 and 5 seconds shorter per patient in the first and second nurses, respectively.

**Conclusions:**

With this modification, the diagnostic power has increased by 22% in the first nurse and 29% in the second nurse. Due to the significant increase in the accuracy of the mSALT (Modified SALT) triage method, this modification can be considered useful and can be used to advance the goals of triage in MCIs.

## Introduction

Since the beginning, human beings have always suffered many financial and human losses from man-made and natural disasters.^1-3^ Based on the United Nations (UN), from 1994 to 2005, the disasters killed 606,000 people and left 4.1 billion injured, homeless, and in need of urgent health care. In addition, 1.25 million are killed and about 50 million are injured in road accidents annually.^4^ From the above statistics, it is clear that there is a need to address this issue; but one of the problems in dealing with these incidents is the lack of a comprehensive definition of Disaster or MCIs.^5^ The World Health Organization (WHO) calls MCIs a situation in which the number of casualties exceeds local medical resources and it requires exceptional emergency arrangements and additional or extraordinary assistance.^5-9^ These situations can be from a road accident to widespread incidents such as earthquakes.^10,11^

In a crisis caused by MCIs and lack of resources, the most important management point is to prevent the deviation of resources from the neediest patients to patients with minor injuries.^12^ This deviation can be prevented by considering the managerial attitude of "greatest goods for the greatest numbers" and to implement it, it is necessary to use a process called triage.^13^ Triage is one of the most effective factors in the fair and proportional distribution of treatment facilities among patients in need^14-17^ and creates an appropriate discipline in the MCIs response process.^18,19^ This concept has been brought from the battlefield to civilian conditions for two centuries ago and has been developed in various forms.^12,13,20^

Here we deal with triage methods in MCIs, which include: a) Primary triage: This type of triage at the scene of the incidents classifies the injured based on the clinical condition.^21,22^ b) Secondary triage: In this process, patients are triaged at the entrance of the Emergency Department (ED) of the hospital or Disaster Medical Aid Center (DMAC) in Widespread incidents, to prevent chaos and deviation of resources.^10,12,23,24,25^ c) Tertiary triage: This type of triage is done by specialist physicians for surgical intervention or special treatment services based on the chance of survival.^21,23^

There are numerous reports of failure and success of using triage methods in MCIs; However, due to the known and unknown variables, its cause is not exactly recognizable.^13^ Undertriage and failure to arrive at the treatment time for patients and even overtriage cause a poor prognosis for patients in MCIs.^13^ Consequently, consistent with the implementation of disaster preparedness processes, action should also be taken to reduce the error rate of triage methods.^11^ Furthermore, to choose a suitable triage method, indicators such as cost of implementation, ability to use, speed of triage, and the possibility of employment should be considered in different numbers of patients and incidents with different conditions.^26^

In a study by Robert et al. (2018), it was found that the highest undertriage in the compared triage methods was related to differentiating the patient with minor injury (green) from the patient with serious injury (yellow). This issue was especially evident in the SALT triage method due to the subjective nature of this step.^11^ To solve this problem, the designers of the SALT, particularly in differentiating the immediate patient (red) from the expectant patient (gray), have recommended that if the triage officer is hesitant to make a decision, he or she can overtriage these patients until a person with higher knowledge and experience arrives.^10^ The design committee of the SALT triage introduced it as a primary and processable method.^13^

Despite the mentioned, Robert et al. (2018) recommended the SALT triage method due to its ability to identify patients in all age groups and chemical and radiological incidents, and on the other hand, called for a more accurate evaluation of this method and modification of existing challenges.^11^ For instance, the Simple Triage and Rapid Treatment (START) triage method was modified and revised after design and presentation to the treatment environment. Due to the variable amount of ambient light, and the effect of substances affecting the arteries, especially in chemical incidents, this criterion gave way to the study of the radial pulse.^27^

In 2015, Faizan et al. by adding an orange group of patients, modified the START triage method. This group consisted of patients with chest pain, shortness of breath, and head trauma whose changes in physiological parameters were not such that the START method categorized them as red. After comparing the two methods on 28 hypothetical patients, it was found that the overall accuracy for the FDNY-START^1^ triage group was 91.2% and for the START group was 87.1% and in five patients, the orange category was 86.3% and 81.5%, respectively. Consequently, it was found that the FDNY-START triage method is more accurate than the common method.^10,17^

In the study of Cooper et al. (2005), to modify and develop the Jump-START triage method algorithm in MCIs for proper triage of victims in the hot zone of the incident or the unavailability of bag valve mask (BVM) in the triage staging area. Based on this improvement, patients who start breathing in the hot zone with the maneuver of opening the airway were initially considered red, and if after being removed from the hot zone and decontamination, their respiratory range is in the range of was more than 40 and less than 20, they were definitely in the red group. Patients who did not start spontaneous respiration after opening the airway were initially considered black, and after exhalation and presentation of two respirations, if spontaneous respiration did not return, they were definitely in the black group. According to the designers, this correction will contribute to the correct triage of patients.

Regarding the unfortunate consequences of triage errors for patients,^13^ and according to the study of Lerner et al. (2008) and Robert et al. (2018) about the challenges of the SALT method and availability of an acceptable criterion (Reference Standard ), this study intends to modify and develop the SALT triage method. Using the criteria in the Reference Standard, in defining the patient of minor and fatal injury and attaching items to the main algorithm, it tried to facilitate decision-making in two subjective steps (differentiation between the green category from yellow and red from gray). The researcher tries to increase the diagnostic power of users and thus the accuracy of this method. Consequently, the current study was done to determine, develop and validate the SALT triage method to facilitate the identification of patients with minor (green category) and fatal (gray category) injuries in MCIs in 2020.

## Methods 

**Study design**

This study is a descriptive cross-sectional approach and using the study of SALT triage method and using the criteria defined in the Reference Standard, with the attachment of items, facilitates identification of patients with minor and fatal injuries.

Structure of the SALT triage method: This method is a five-level method that performs the general classification and individual evaluation of the triage process in two stages. In the general classification stage, the injured of the accident are classified into three general groups by voice command. The first group: patients who can walk and move to the designated place; Group 2: Patients who shake their hands with a voice command. Group 3: Patients who, due to the deterioration of the clinical condition, are unable to follow a simple command or have life-threatening conditions; this group of patients is the priority for individual evaluation and rescue intervention.

**Figure 1:**
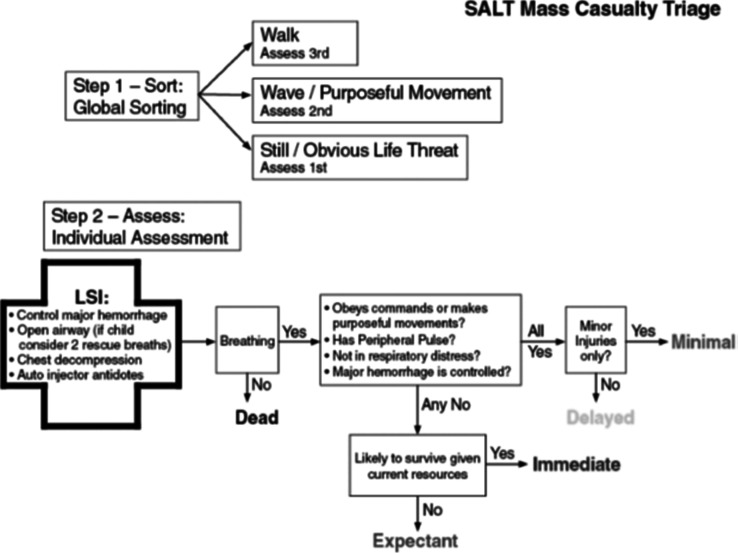
SALT Triage Algorithm. ^10,18^

Individual evaluation phase: First, if necessary, the therapeutic intervention includes: opening the airway and (with two assisted breathing for children), control of life-threatening bleeding, use of self-injection antidotes, and needle decompression for the patient. The triage officer then evaluates the patient by rapidly evaluating physiological parameters including respiratory quality, radial pulse palpation, level of consciousness. If all the above characteristics were normal, the triage officer would place the patient in the green or yellow category based on his or her diagnosis of the extent of the damage. However, in case of disturbance in any of these indicators or uncontrollable bleeding, the triage officer, by estimating the available facilities and the patient's chance of survival, places the patient in the category of gray or red (Figure 1).^10,18^ The mentioned items have been attached to the SALT triage algorithm to facilitate the identification and differentiation of patients in the subjective steps

**Definition of minor injuries (outpatient or green category):**

- An organ injury that has no obvious signs of dislocation (displacement, swelling, and severe tenderness), fracture (crepitus, displacement, severe swelling, and tenderness), or vascular injury (paleness and lack of distal pulse).

- Soft tissue damage that only needs a simple wound repair (such as a simple skin suture).

- Chemical or radiologic exposure in a patient with no obvious symptoms that only requires superficial decontamination.

**Definition of fatal injuries (Expectant or gray category):**

- Full-thickness burns over 80% of the total body area.

- Trauma-induced brain injury with symptoms of agonal respiration.

- Uncontrolled hemorrhage that resulted in cardiac arrest (lack of pulpable pulse and CPR is being presented).

- Chemical exposure with an agonal respiration or cardiac arrest (lack of pulpable pulse and CPR is being presented) after any administration of the available antidote.

- Radiological exposure with trauma or burns, where the patient has any of the symptoms of nausea, seizures, agonal respiration, or cardiac arrest (lack of pulpable pulse and CPR is being presented).

**Participants**

There were two nurses, one female with five years of work experience and one male with eight years of work experience. None of them had been trained in SALT triage method before this study, and had not par-ticipated in any MCIs in the past. The population under study was the triage nurses of the ED, that were select-ed by sampling at convenience.

100 studied patients were triaged by two mentioned nurses at two different times (two months apart) and methods (SALT then mSALT). The scenario of the patients under study was prepared on the basis of the medical information of the traumatic patients admitted for a certain period of time in the ED of Imam Houssine (AS) Hospital. The hospital is general and educational hospital with 557 active beds in Tehran, Iran. Nurses working in the ED of the hospital and patients who consented to disclose their medical information were added to the study and otherwise excluded from the study. 

**Data collection**

At first, author has studied and analyzed the defining terms of expectant (gray) category and outpatient category (green) in the Reference Standard. The researcher then selects the definitions that are related to clinical signs and can be understood and identified by rapid clinical evaluation, and after editing, appends them to the algorithm in the form of footnotes. The validity of the instrument was confirmed qualitatively and formally by 25 relevant experts using the content validity method.

In the next stage, two research colleagues unaware of the purpose of the research, using the medical data collection form^3^, medical history, and vital signs of trauma patients admitted to the ED of Imam Hossein (A) Hospital in all age groups were collected in 96 hours. These patients were then categorized into gray, red, yellow, green, and black categories based on Reference Standard, and scenarios containing a brief history of the patient's chief complaint, the mechanism of injury, and the physiological parameters required to identify patients were written. Ten experts in this field confirmed the face and content validities of these scenarios.

The simulated scene was designed for patients who were able to walk standing and the rest lying on the floor and children under five years in the arms of the mother, in the backyard of the hospital and scenarios in the sheet installed on the chests of the mannequins. After completing the SALT triage training, two nurses in the ED of Imam Hossein Hospital were asked to triage 100 simulated patients. The results were evaluated for accuracy with a predetermined classification by the Reference Standard. Triage time was also measured with an exact timer by two research colleagues, from the beginning of reading the first patient scenario to the installation of the last patient triage label. The SALT triage method was taught during a class for one hour. Solve the problems with 10 sample scenarios and questions and answers, for two emergency nurses of Imam Hossein (AS) Hospital was done. They were then asked to triage 100 simulated patients.

The results were evaluated for accuracy with a predetermined classification by the reference standard.

The triage time was also measured by two research colleagues with a precise timer, from the beginning of reading the first scenario of the patient to the installation of the last patient triage label.

This test was conducted again after one month with the training of the modified SALT triage method and the data obtained from it were compared with the data of the previous exercise in terms of speed and accuracy.

**Tools**

The data collection form used for medical record review published in the 2019 study by Courtney et al. was used to collect medical information of patients admitted to the ED and this information was used as a model for designing a simulated patient scenario.

The Consensus-based Golden Standard for Mass Casualty Incident Triage Systems was used as a basis for comparing the two common and modified SALT triage methods and the reference for preparing items attached to the SALT triage method algorithm.

**Statistical analysis**

The accuracy of patients' triage was estimated using sensitivity and specificity and compared with each other as a percentage. The time spent in the triage process in each group was described descriptively with each other and the recommended duration of triage in MCIs was compared.

## Results

Table 1 shows the distribution of injuries in the 100 studied patients. Table 2 presents the demographic characteristics of studied nurses. 

**Table 1:** Distribution of injuries in the studied patients.

Types of injuries	Number	Percentage
Head trauma	24	24%
Extremities’ trauma	32	32%
Chest trauma	6	6%
Soft tissue injuries	6	6%
Abdominal trauma	1	1%
Multiple trauma	15	15%
Burning	1	1%
Shock and bleeding	2	2%
Cardiac arrest	5	5%
None trauma	2	2%
Spinal and pelvic trauma	6	6%
Total Patients	100	100%

**Table 2:** Demographic characteristics of nurses.

Variables	Nurse 1	Nurse 2
Gender	Male	Female
Work experience	8 year	5 year
Mass Casualty Incident experi-ence	NO	NO
SALT triage past training	NO	NO

**Nurse number one, using SALT**

The number one nurse with the usual SALT triage method, with 5% over triage, 33% under triage, and 62% overall accuracy. In general, in over triage, 4% of the triage of patients with minor injury (green) was in the category of patients with serious injury (yellow) and 1% of the triage of the patient with fatal injury (gray) was in the immediate category (red). In total under triage, 23% of the patient's triage was yellow in the green category, 8% was related to the red patient triage in the yellow category, and 2% was related to the gray patient in the red category. More detailed analysis shows that the accuracy of triage in the category of patients was 82% in green, 57% yellow, 23% red, 87.5% gray, and 100% in the black category (Table 3). The triage rate was also measured for an average of 25 seconds per patient (Table 4 ).

**Table 3:** Comparison of the accuracy of nurse triage number one and two in comparison with the Reference Standard using SALT triage method in 2021.

Nurse 1							
	Reference Standard	Green	Yellow	Red	Gray	Black	Total
SALT							
Green	18	23					
Yellow	4	30	8				
Red			3	1			
Gray			2	7			
Black						4	
Total		22	53	13	8	4	100
Accuracy (consistency)Time: 2520 Second		82%	57%	23%	87.5%	100%	62%
Nurse 2							
	Reference Standard	Green	Yellow	Red	Gray	Black	Total
mSALT							
Green	13	25					
Yellow	9	28	8				
Red			5	3			
Gray				5			
Black						4	
Total	22	53	13	8	4	100	
Accuracy (consistency)Time: 3500 Second	59%	53%	38.5%	62.5%	100%	55%	

**Table 4:** Comparison of the average speed of first and second nurse triage using SALT and mSALT triage methods in 2021.

Participants	Groups	Total time (second)	Average triage time for each patient
Nurse 1	SALT	2520	25 seconds
	mSALT	2100	21 seconds
Nurse 2	SALT	3500	35 seconds
	mSALT	3000	30 seconds

**Nurse number two, using SALT**

The second nurse was using the usual SALT triage method, with 12% overtriage and 33% undertriage, and 55% overall accuracy. Of the total overtriage, 9% of the green patient's triage was in the yellow category and 3% was related to the gray patient triage in the red category. Of the total overtriage, 25% of the yellow patient's under triage was in the green category and 8% was related to the red patient's under triage in the yellow category. The detailed analysis of the data reveals that the accuracy of triage in the category of green patients was 59%, yellow 53%, red 38.5%, gray 62.5%, and black 100% (Table 3). Triage speed was also measured for an average of 35 seconds per patient (Table 4 ).

**Nurse number one, using mSALT**

Nurse number one via the modified SALT triage method (mSALT), was without over triage, 8% under triage, and overall accuracy was 92%. Under triage was related to the patient's red triage in the yellow category (Table 5). The triage rate was measured for an average of 21 seconds per patient (Table 4). The correction made in the SALT triage method could reduce the overall accuracy of the first nurse triage by 22%. In a more detailed analysis of these data, it was found that in the classification of patients, the category of green has improved by 18%, yellow by 43%, red by 15%, and gray by 13%. The triage speed was measured for an average of 21 seconds per patient. It showed that the use of the mSALT method was faster by an average of 4 seconds per patient.

**Table 5:** Comparison of the accuracy of nurse triage number one and two in in comparison with the Reference Standard in 2021.

	Reference Standard	Green	Yellow	Red	Gray	Black	Total
SALT							
Green		22					
Yellow		53	8				
Red			5				
Gray				8			
Black					4		
Total		22	53	13	8	4	100
Accuracy (consistency)		100%	100%	38.5%	100%	100%	92%
Time N1: 2100 Second							
Time N2: 3000 Second							

**Nurse number two, using mSALT**

Nurse number two using the mSALT triage method was without over triage, 8% under triage, and 92% overall accuracy. All of the under triage was related to the patient's red triage in the yellow category (Table 5 ). The triage rate was measured for an average of 30 seconds per patient (Table 4). The improvement made in the SALT triage method could reduce the overall accuracy of the second nurse triage by 29%. In a more detailed analysis of these data, it was found that the category of patients, green, 41%, yellow, 47%, red, 0%, and gray, 38%, were improved. The triage speed is accelerated by an average of 5 seconds per patient.

## Discussion

This study aimed to determine, develop and validate the SALT triage method to facilitate the identification of minor and fatal injuries in MCIs.

The data show that the first nurse using the usual method was able to correctly identify more than three quarters of green patients, more than half of yellows, one quarter of reds, more than three quarters of grays, and all blacks. In the second nurse was more than half of the green patients, half of the yellows, more than one third of the reds, more than half of the grays, and all blacks.

After using the mSALT triage method, both nurses were able to correctly identify all patients in black, gray, yellow, green, and more than one third of patients in the red category. In other words, this correction was able to include 22 patients who were mistakenly triaged by the first nurse, of which four were in the green category, 23 were yellow, two were red and three were gray. The second nurse could place 29 mistakenly triaged patients in the appropriate category, of which 9 were green, 25 were yellow and three were gray. Of these, only eight red patients, equal to more than half of the patients in this category, remained in the yellow category (under triage). The reason for this was the lack of signs of severe injury in these patients in their physiological parameters (peripheral pulse, respiration, and level of consciousness), which was related to the structure of the SALT algorithm and the step of identifying immediate patients and not related to steps that the current study intends to correct it.

Faizan et al. (2015) were able to increase the accuracy of the START triage method by 4% in overall accuracy and 5% in the target category (orange color). This alteration is approximately one-fifth the increase in overall accuracy as a result of the modification of the SALT triage method and can be a good indicator of the effectiveness of the correction in the structure of the two triage methods in the Faizan study compared to the present study. This issue can be attributed to the structural differences between the two methods, the amount and manner of corrective intervention and the quality of the steps intervened in the present study, and its considerable flexibility.

Data from Courtney et al.’s (2019) study Show that the SALT triage method was able to identify nearly half of red patients, more than three-quarters of grays, nearly half of yellows, and more than three-quarters of greens and all blacks correctly. The accuracy of the mSALT triage method, compared to the results of the study, revealed a significant difference, especially in the green, yellow and gray categories. In the red category, in both studies, nearly half of the patients were correctly identified.

As formerly explained, the error in the present study was due to the structure of the SALT triage method. Nevertheless, in Courtney et al.’s research, three-quarters of the errors were related to the classification of red patients in the expectant category, which indicated the inability of the triage officer to distinguish between red and gray patients. This fact reveals the challenge of the SALT triage method and demonstrates the advantages of the mSALT method over the conventional method.

The SALT triage method in the study of Salvatore Silvestri et al. (2017) could correctly identify approximately three quarters of patients in the red, yellow, green, and all black patients. The accuracy of the mSALT triage method is significantly higher in the green and yellow categories. Nevertheless, in the red category, the accuracy of the usual method in the Sylvester study is higher than the mSALT method. Since detailed information on the amount of overexpression and underarms in the red category has not been reported, it is simply not possible to understand the cause of this issue. However, this concern may be due to the number of patients, the type of injuries, the unrealistic medical history of patients, and especially the inclusion of gray patients in this study. The explanation is that not including the category of gray patients automatically eliminates the possibility of error in differentiating patients from this category from the red category, and as a result, by reducing the probability of error, the accuracy in identifying patients in this category increases falsely.

Robert et al.’s (2018) study revealed that the SALT triage method was able to identify correctly three-quarters of red patients, none of the patients in grays, one quarter of yellows, almost all greens, and all blacks. The data indicate that 37 yellow patients were classified in the green category, 2 green patients in the yellow category, and only gray patients in the red category. Compared with the data obtained from the mSALT triage method, it was found that the classification of patients in the subjective stages, the differentiation between which is a challenge for the triage officer, was so excessive that the author himself pointed out the existing challenge and asked for a solution. 

However, the higher accuracy of the usual SALT method in the category of red can be attributed to the small number of patients in this group (four times less) than the present study and the type of injuries to patients. Moreover, based on the supervisor's opinion in differentiating these patients, these classifications can change with the change of supervisor and as a result, affect this comparison as well.

With the improvement, the triage speed of the first and second nurses was shortened by 4 and 5 seconds per patient, respectively. This can be due to the systematic process of patient evaluation and facilitating nurses' decisions. The measured time, both in the modified and non-modified methods, was within the recommended and acceptable range compared to the study of Sylvester et al. However, compared to the study by Courtney et al., Faizan et al., and Robert et al., who did not consider the evaluation of this important indicator, is an advantage.

By using the history of real patients in all age groups in an acceptable number of patients and simulating the scene of patients rushing to the ED, this study aimed to study the use of this tool in conditions close to MCIs. In addition, not like previous studies, this study has precisely analyzed the degree of accuracy in various categories and statutes of the errors.

**Limitations**

Studying simulated patients whose histories are written in the form can affect the rapid physical examination process to determine the extent of the patient's injury. On the other hand, takes the possibility of making a mistake in evaluating the real patient from the triage officer. Furthermore, the actual condition of the MCIs and the resulting stress and disorder can reduce the speed and accuracy of the triage officer. Non-trauma patients were not included in the study patients due to the lack of identification of non-trauma patients by Reference Standard. Studying the usual patients admitted to the hospital can also be inconsistent with the various instances that are likely to occur.

## Conclusion

Regarding the significant upsurge in the accuracy of the mSALT triage method, this adjustment can be considered useful and can be used to advance triage goals in MCI. 

This improvement can serve as a guide or suggestion in the service of triage in MCI, and if a person with high medical knowledge and experience, depending on the type and extent of the accident and the number of injured and access to resources, decides to apply flexibility in triage, can be set aside and operate according to local protocols and special conditions.

**Acknowledgement**

We like to express our appreciation to nurse’s work in Imam Hussein hospital affiliated to Shahid Beheshti University of Medical Sciences (SBMU), Tehran, Iran. 
